# Surgical planning for future endovascular aortic arch interventions

**DOI:** 10.1016/j.xjse.2026.100154

**Published:** 2026-10-06

**Authors:** Ryaan EL-Andari, Pierre Dionne, Mathew Cwinn, Joel Gagnon, Michael C. Moon

**Affiliations:** a Division of Cardiac Surgery, Department of Surgery, University of Alberta, Edmonton, Alberta, Canada; b Division of Cardiac Surgery, Department of Surgery, Royal Columbian Hospital, New Westminster, British Columbia, Canada; c Division of Vascular Surgery, Department of Surgery, University of Alberta, Edmonton, Alberta, Canada; d Division of Vascular Surgery, Department of Surgery, Royal Columbian Hospital, New Westminster, British Columbia, Canada

**Keywords:** aortic arch, aortic dissection, endovascular arch repair

**Figure:**
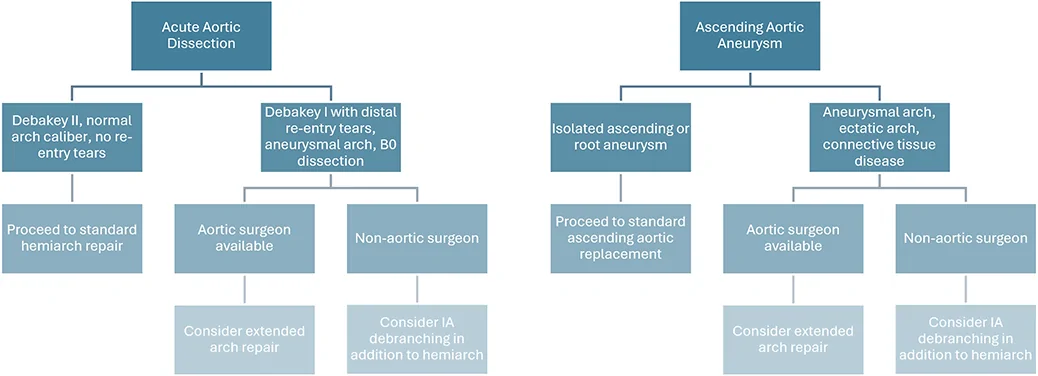
Flow diagram for patient selection for aortic arch debranching considerations.

**Central Message**

Consideration of future reinterventions at the time of initial open aortic surgery will help to facilitate endovascular arch interventions that may be required in the future.

Endovascular arch repair (EAR) has become increasingly versatile in the preceding decades, advancing from interventions on isolated segments of the descending thoracic aorta to endovascular arch interventions and even emerging cases of endo-Bentall procedures.^1^, ^2^, ^3^, ^4^, ^5^ Although EAR is largely limited to high surgical risk cases, the indications for EAR are expanding. Furthermore, hybrid procedures combining surgical and endovascular options are being more frequently used.^1^, ^6^, ^7^ Ethics approval or consent was not required because no patient data or information was included in this study.

Acute type A aortic dissection (ATAAD) traditionally has been associated with high rates of perioperative morbidity and mortality. As a result, the focus of research has traditionally been on improving the short-term outcomes of these patients. Advancements in surgical technique, endovascular devices, and postoperative management of patients with aortic dissection have reduced the rates of short-term mortality for these patients.^2^, ^5^, ^8^, ^9^, ^10^, ^11^, ^12^, ^13^ Patients with ascending aortic aneurysm less frequently present for emergency surgical repair; however, similar advances such as frozen elephant trunk (FET) devices have revolutionized the surgical management of these patients.

With more patients surviving their initial surgical intervention, there has been a recent shift in focus toward mid- to long-term outcomes of patients with aortic disease, as well as greater consideration for aortic remodeling and reintervention. Additionally, with EAR becoming more frequently used, patients who are at high risk for reoperative arch procedures have a wider range of options to facilitate reintervention. With increasing use of an EAR likely to continue, optimization of the initial surgical repair to facilitate EAR is of considerable importance. Several features of the initial surgical repair of ascending aortic disease may help to facilitate or even hinder future EAR. We describe these key details in an attempt to illustrate the optimal approaches to surgical repair of ascending aortic disease to facilitate EAR.

## Current Approaches to Endovascular Aortic Arch Intervention and Reintervention

Several off-the-shelf devices are available and custom-made devices have been described in the literature for EAR.^1^, ^2^, ^5^ In general, access is needed to the branch vessels of interest and to the femoral arteries for deployment of the arch device. Upper-extremity access and, in some cases, carotid access are also frequently required. The endovascular graft is advanced into the ascending aorta and deployed in zone 0 above the sinotubular junction (STJ) or in Zone 1. The branches are then catheterized, and covered stents are deployed with landing zones in the branch vessels and the arch graft restoring perfusion to the head vessels.^5^, 14.], 15, 16 Another approach is in situ laser fenestration in which a laser is used to create perforations in the arch graft postdeployment through which covered stents can be deployed into the head vessels.^13^, ^17^

Alternatively, a hybrid approach with surgical branch vessel transpositions or bypasses may be used to ensure perfusion of the head vessels before EAR and coverage of the arch.^1^, ^7^, ^15^, ^18^, ^19^, ^20^ Sternotomy can still be used to facilitate replacement of the ascending aorta, deployment of a FET device, and debranching of the head vessels proximally to allow for the landing of an aortic arch device, whereas either a sternotomy or cervical incision can be used to allow access for head vessel bypasses.^1^, ^6^, ^19^

### Endovascular Arch Repair Configurations

The general configuration can be a 1, 2, or 3-branch configuration. In the 1-branch configuration, a single branch is used to perfuse the innominate artery (IA), and the remaining head vessels are perfused via carotid-carotid and carotid-subclavian bypasses. In the 2-branch approach, the IA and left common carotid (LCC) artery or left subclavian artery (LSA) are reimplanted, with only the LSA requiring bypass. A 3-branch technique is also possible. This involves landing the proximal aspect of the device in the ascending aorta, often in the setting of a prior ascending repair, with 3 retrograde branches for the IA, LCC, and LSA.

The impact of these techniques on future endovascular interventions is that although the 1-branch approach simplifies future EAR that requires cannulation of this graft, this approach depends on the patency of the bypasses and has the added morbidity of complex cervical debranching. The inverse is true for the 2-branch technique, because future EAR may be more complicated, but there is a reduced risk from cervical debranching because these patients do not require a carotid-carotid bypass. The 3-branch option involves the most endovascular complexity and requires specific anatomy for procedural feasibility, but avoids the need for additional neck incisions and bypasses. The selection among these 3 endovascular approaches should be individualized to the patient.

### Outcomes After Endovascular Aortic Repair

Numerous studies have been published on the outcomes after primary EAR in most cases. A recent systematic review by Basha and colleagues^8^ pooled outcomes from patients undergoing EAR, with devices landing in Zone 0. The frequency of the techniques used in the included studies was 10% single-branch, 70% 2-branch, and 19% 3-branch. This study identified pooled rates of mortality at 16%, cerebrovascular accident rate of 14%, perioperative myocardial infarction of 4%, and pooled rates of endoleak at 13%.^8^ It is important to note that the results reported in this review were heterogeneous, in part due to the initial experiences at some centers and the associated learning curves, as well as the advancements in device technology. The outcomes of EAR will likely continue to improve over time as experience, patient selection, and device design improve.

## Considerations at the Time of Initial Aortic Intervention for Future Endovascular Arch Repair

### Patient Selection

Not all patients presenting for ascending aortic repair, whether with ATAAD or aneurysm, are at the same risk for requiring future arch reinterventions (Figure 1). Patients with DeBakey II aortic dissections or isolated ascending or root aneurysms can have a curative repair with an ascending aortic replacement (±hemiarch). Patients with ATAAD extending into the aortic arch (DeBakey I aortic dissection), particularly those with re-entry tears in the aorta distal to the repair or in the head vessels, patients with B0 dissections, and those with aneurysmal or ectatic arches, are at the highest risk of requiring reintervention and should be considered for modification of the index surgical repair if isolated to the ascending aorta. Patients who have extensive arch disease and are treated by an aortic surgeon may undergo a total arch repair with or without FET, which would obviate the need for EAR but does carry additional risk such as spinal cord injury and is not indicated for all patients with disease extending into the arch. The patients who may benefit from the following considerations are those who are at risk for progression of aortic arch disease but who do not undergo extended arch repairs either due to nonaortic surgeons performing the case or instability necessitating a simpler initial repair.

**Figure 1:**
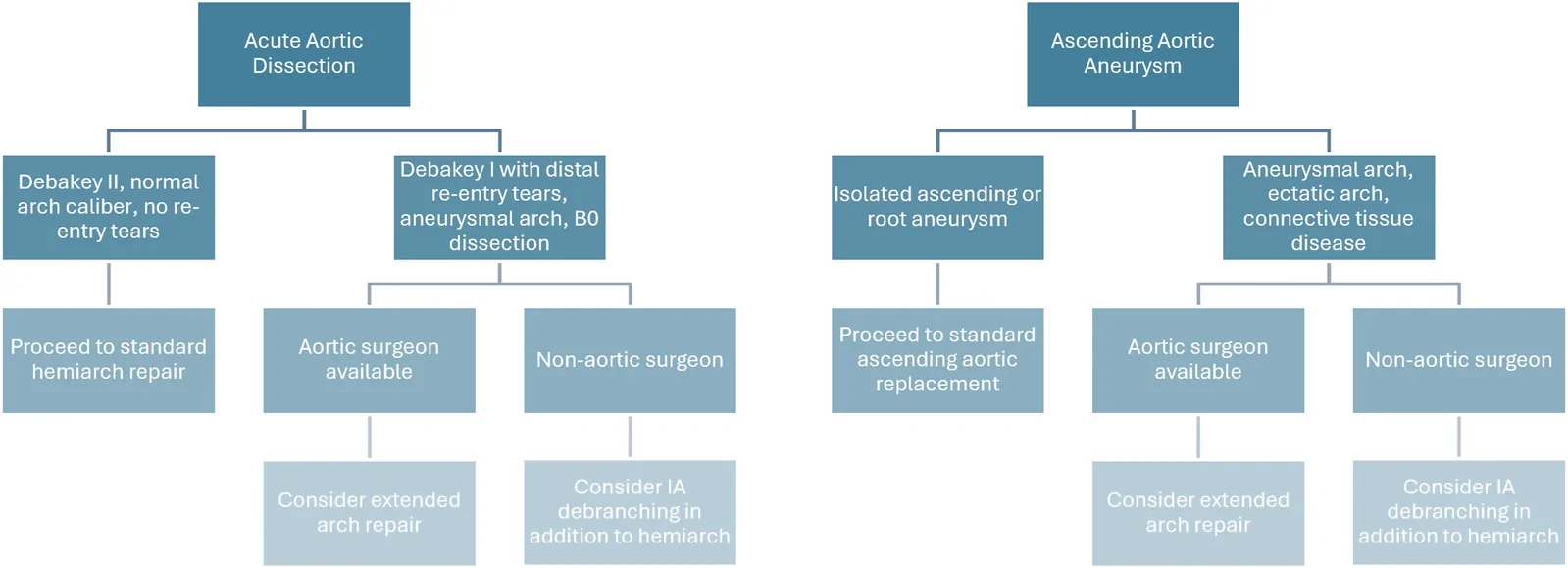
Flow diagram for patient selection for aortic arch debranching considerations. *IA*, Innominate artery.

### Graft Length and Location

The length of the hemiarch repair is of paramount importance in facilitating future EAR and providing an adequate landing zone. Current off-the-shelf endovascular arch devices such as the Gore or Cook devices require a 40-mm proximal landing zone. Although not all devices may require a landing zone at this length, it is not possible to predict which patients will require EAR and with which devices. A proximal sealing zone of approximately 40 mm would provide a suitable landing zone in most EAR cases.^5^, ^19^, ^21^ Ensuring there is an adequate length of Dacron proximally between the head vessels and the STJ will ensure adequate length is available for a proximal landing zone. At the time of the initial procedure, the surgeon must take note of the most proximal location of the aortic pathology and ensure that there is at least 40 mm of graft or healthy aorta to act as a landing zone for future interventions (Figure 2).

**Figure 2:**
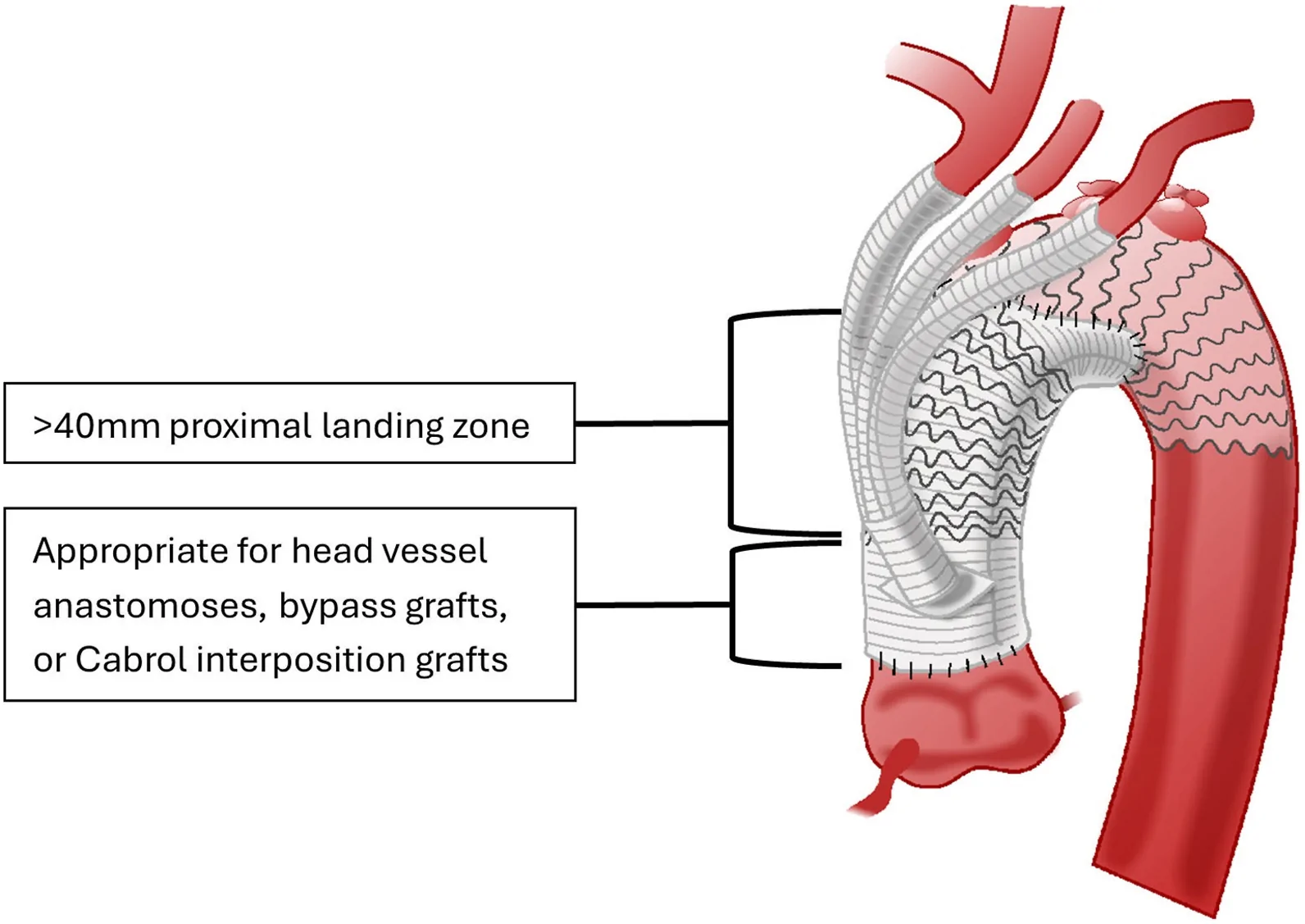
Endovascular arch repair in a previous hemiarch repair with debranching of the head vessels proximally, indication of the 40-mm proximal landing zone, and optimal area for anastomoses and bypass grafts.

Caution must be taken when measuring the graft for the ascending aortic replacement, because excess length is also undesirable. Even if 40 mm of ascending aortic graft is present, if there is excess graft length, there will be angulation or kinking of the graft reducing the pushability of the delivery device, making it more difficult to maneuver and land an endovascular stent graft in this region, in addition to increasing the risk of postoperative endoleaks.^5^, ^19^, ^21^ The optimal length of graft for replacement will vary from one patient to the next, but can be achieved by resection of the ascending aorta from the STJ to the IA and ensuring the final length is measured with the graft being filled or stretched to prevent oversizing and laxity of the graft. In cases where graft length is too long and kinking is apparent, an alternative to taking down the graft and redoing the anastomosis is to place pledgeted sutures at the greater curve to plicate the graft and reduce the length of the greater curve, reducing angulation.

### Coronary Artery Location and Management

Consideration of branch vessels, both native and reconstructed, is essential for endovascular repairs. Although the traditional landing zone of EAR is within zone 0 and distal to the STJ, coronary location is still an important consideration. In patients undergoing aortic root surgery, reimplantation of the coronary buttons should be performed in the most anatomically suitable location. This is almost always in the aortic root, which should not be at risk of coverage by an EAR and should not preclude this approach.

In contrast, patients who are undergoing coronary artery bypass grafting (CABG) surgery are at risk if the proximal anastomoses of the bypass grafts are relatively high on the ascending aorta. In the unlikely scenario that a Cabrol interposition graft is required, it is often placed above the STJ, increasing the risk of coverage during endovascular stent deployment in the ascending aorta (Figure 3). In patients undergoing CABG or aortic root interventions at the index surgery with the potential for future arch interventions, consideration should be made for the choice of placement for the aortic anastomoses more proximally on the ascending aorta, just above the STJ, because this will reduce the risk of coverage by the EAR.^5^, ^22^

**Figure 3:**
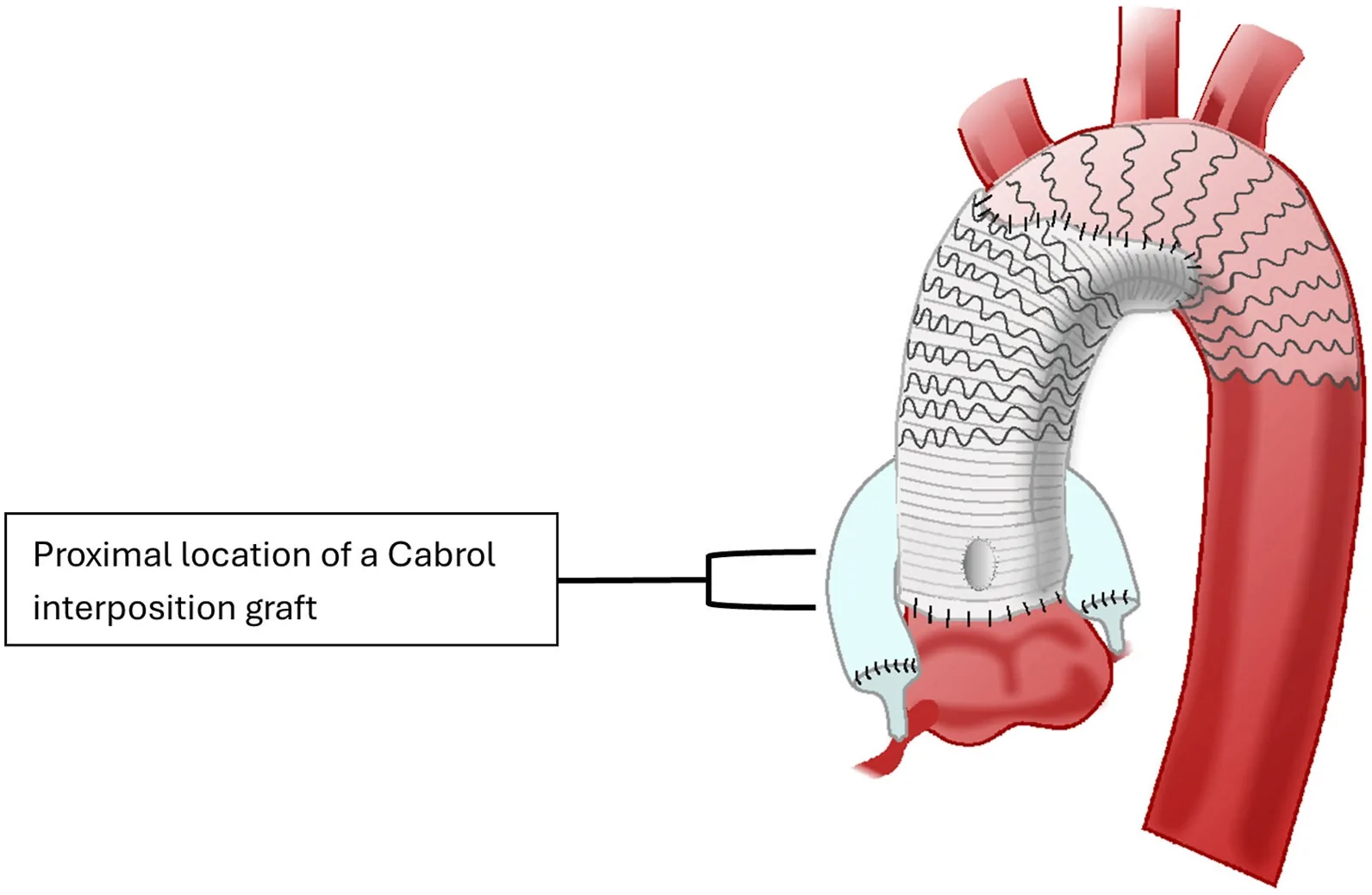
A Cabrol interposition graft illustrating the proximal location of the graft above the STJ allowing sufficient space for the proximal landing zone.

### Head Vessel Location and Management

The location of the head vessels will vary between patients and depends on native anatomy as well as any previous arch interventions. In patients with native aortic arch anatomy that have undergone hemiarch replacement, head vessel management will involve the use of a branched graft using a custom premade device or modified device, in situ laser fenestration, and potentially some combination of head vessel bypass as previously mentioned.^1^, ^6^, ^7^, ^15^, ^18^, ^19^, ^20^

Debranching of the aortic arch at the time of initial surgery allows for proximalization of the head vessels, with or without the use of a FET device to complete the aortic arch repair. This technique has been described in several variations and allows the distal anastomosis of the aortic repair to be placed between zones 0 and 3.^23^, ^24^ Complete debranching of the aortic arch allows for EAR with a covered graft without the need for branches or fenestrations; however, this is a complex procedure that is most often performed by aortic surgeons at the time of total arch replacement.^6^ This can be done as part of the index aortic arch repair or as part of a hybrid repair before EAR.^6^ In cases where the aortic arch has been partially or completely debranched, the location of the anastomoses of the head vessels plays a key role in guiding EAR. With aortic arch debranching, a single trifurcated branched graft may be used to consolidate the head vessel anastomoses to one location in the ascending aorta. If this graft is placed proximally in the ascending aortic graft, this reduces the length of the proximal landing zone but may leave a sufficient landing zone such that a covered arch graft may be deployed, repairing the arch without further intervention required. However, this approach would make any future cardiac surgical procedures such as CABG or aortic valve interventions significantly more challenging. If the branch anastomosis is performed closer to the distal anastomosis of the ascending aortic graft, this may allow for access to the branch vessel and stenting of the branch allowing for the landing zone to be proximal to the head vessel anastomosis and providing a longer proximal sealing zone, although this will increase the complexity of the endovascular repair. Anastomoses placed in the middle portion of the graft may limit the proximal landing zone and preclude future EAR.

Several points are important to consider regarding planning for head vessel debranching at the time of a hemiarch operation. The IA often features a wide and short segment before bifurcation, is not favorable for endovascular interventions, and is often the most challenging anatomic factor in EAR. Even with these factors in mind, previous studies have described techniques for incorporating the IA to EAR and have found that approximately two-thirds of patients had acceptable anatomy for EAR, although this leaves a significant proportion with anatomic contraindications based on the IA.^25^^,^^E1^ In contrast, the LCC often has a long segment between the origin and bifurcation that helps to facilitate stenting. Stenting of the LSA depends on anatomy and prior interventions. In patients with a nondominant vertebral artery and no previous CABG procedures, stenting of the LSA is often feasible. In cases of a dominant vertebral artery or a previous CABG using an in situ left internal thoracic artery graft, stenting of the LSA may not be feasible.

With these factors in mind for a zone 0 or 1 hemiarch repair, where an extended arch repair is not required or is not safely accomplished in cases where a nonaortic surgeon is managing an ATAAD, debranching of the IA provides several benefits. These benefits include facilitating future EAR by extending the proximal landing zone, excluding the IA in case of branch re-entry tears to prevent false lumen perfusion, and reducing the risk of unsuitable IA anatomy precluding EAR. In contrast to total arch replacement and debranching of all of the head vessels, debranching the IA is relatively simpler and feasible for cardiac surgeons without formal aortic training while leaving the LCC and LSA, which are more likely to be managed endovascularly or via bypasses if required. Debranching of the IA during an open aortic repair involves ligating the IA and anastomosing a graft in an end-to-end fashion to the IA and an end-to-side fashion to the ascending aorta; this can be done during rewarming and reperfusion with the IA can be clamped distally and a side-biting clamp placed on the ascending aortic Dacron graft, allowing for 2 anastomoses that do not significantly increase the complexity of a hemiarch repair or add to crossclamp times.

## Discussion

The field of aortic surgery has continuously evolved over the preceding decades, incorporating novel techniques and devices for the management of aortic disease. This has resulted in improved outcomes with reduced mortality and increased long-term survival,^11^, ^12^, ^13^ in addition to the finding that up to 20% of patients who have undergone ATAAD repair may require reintervention.^E2^ Consequently, the focus of ATAAD outcomes has begun to shift away from perioperative results to long-term outcomes, such as aortic remodeling and reintervention.^E3^ With a subset of patients with ATAAD or aortic aneurysm requiring repeat aortic intervention being of high surgical risk, reoperative EAR is increasingly used.

Several considerations at the time of initial aortic intervention and subsequent endovascular repairs that may hinder or facilitate reintervention have been highlighted. At the time of initial ascending aortic intervention and hemiarch repair, several methods can be used to optimize conditions for potential future endovascular arch interventions. In the cases of CABG or Cabrol procedure, ensuring implantation of these grafts as proximally as possible will reduce the risk of coverage by future endovascular repairs. For ATAAD, a hemiarch repair may still allow for endovascular arch reinterventions in patients with appropriate anatomy. Although zone 0 hemiarch remains the minimal intervention required for ATAAD, there should be consideration of future interventions because these patients may experience adverse remodeling and require reintervention. The addition of head vessel debranching, at minimum of the IA, may help to optimize endovascular reintervention with minimal increases in surgical complexity and should be considered in cases of ATAAD.

The suggestions for surgical planning at the index repair for future endovascular repairs are based on providing the optimal anatomy to accommodate a range of EAR, recognizing the individual approaches and devices may require differing sets of conditions for suitability. Although the principles outlined in this article are based on principles of endovascular arch interventions, further validation of the proposed interventions will be required with published clinical experiences and trials.

## Conclusions

Endovascular aortic arch interventions are increasing in frequency. Patients undergoing ascending or aortic arch replacement are at risk for adverse remodeling and required reintervention. Several considerations at the time of the index surgical repair may help to facilitate future endovascular reinterventions. In general, ensuring appropriate ascending aortic graft length reduces the risk of an inadequate proximal landing zone or graft angulation. Implantation of proximal anastomoses of CABG or Cabrol interposition grafts will reduce the risk of coverage by EAR. Finally, there should be particular attention paid to the head vessels, because location of reimplantation may impact proximal landing zone length and complexity of future reinterventions.

## Data Availability Statement

No datasets were created in the production of this article.

Ethics approval and consent were not required because no patient data or information was included in this study.

## Conflict of Interest Statement

The authors reported no conflicts of interest.

The *Journal* policy requires editors and reviewers to disclose conflicts of interest and to decline handling or reviewing manuscripts for which they may have a conflict of interest. The editors and reviewers of this article have no conflicts of interest.
